# Effect of fermentation of chosen vegetables on the nutrient, mineral, and biocomponent profile in human and animal nutrition

**DOI:** 10.1038/s41598-022-17782-z

**Published:** 2022-08-04

**Authors:** Piotr Kiczorowski, Bożena Kiczorowska, Wioletta Samolińska, Marek Szmigielski, Anna Winiarska-Mieczan

**Affiliations:** 1 Department of Biological Bases of Food and Feed Technologies, University of Life Sciences in Lublin, Głęboka Street 28, 20-612 Lublin, Poland; 2 Institute of Animal Nutrition and Bromatology, University of Life Sciences, Akademicka Street 13, 20-950 Lublin, Poland

**Keywords:** Metals, Fats, Proteins, Nutrition, Plant sciences, Health care

## Abstract

In the present study, the dry matter, crude ash, crude protein, ether extract, and energy, macro- (Na, K, Ca, Mg, P), micro- (Zn, Cu, Fe) minerals, heavy metals (Pb, Cd), vitamin C, A, carotene, and phenolic content were determined in chosen raw and fermented vegetables. The dietary intake of several macro- and microconstituents per one serving (100 g or humans and animals: ducks and pigs) was calculated. The fermentation process was found to reduce water and increase fat content in the vegetables. Lower levels of vitamin C and phenols were also found in the fermented vegetables. The vitamin A and carotene content in the fermented carrots and peppers were increased in comparison with the raw vegetables. The fermentation process decreased the concentration of some basic nutrients, mineral content, vitamins C and A, and phenols. Broccoli, peppers, and red beet had the highest levels of the analyzed nutrients and bioconstituents. The fermentation process is regarded by nutritionists as beneficial to human health. The addition of fermented plants is recommended in animal nutrition as well. This process modifies the chemical composition of preserved vegetables, e.g. it reduces the concentration of dietary fiber, and brings favorable effects in poultry and pig nutrition.

## Introduction

Lactic acid fermentation is a food preservation process carried out by lactic bacteria. It is believed that the first human experience with fermented food was accidental, and the introduction of such products to diet was associated with the need to improve the durability and storability of food in periods of limited availability. Currently, the lactic acid fermentation process is used not only for preservation purposes but also for manufacturing new processed food with health-enhancing and economic values^1^. Nowadays, fermented food is consumed in almost all countries of the world, although the type of raw materials and the fermentation methods are often specific for geographical regions or countries (Table 1). Many types of vegetables or fruits can be pickled, e.g. broccoli, cauliflower, carrots, onions, garlic, white radish, tomatoes, cucumbers, cabbage, green beans, beet greens, red beet, eggplants, peppers, and even strawberries, plums, pears, and olives^2,3^.

**Table 1:** Lactic acid bacteria involved in the fermentation of selected vegetables^2,13,16^.

Vegetable	Main lactic acid bacteria involved	Country of traditional human and animal consumption of fermented products
Broccoli	*L. plantarum, L. casei, Leuc. pseudoplantarum, L. fermentu, P. pentosaceus*	Asia, Europe
Carrot	*L. plantarum, L. paracasei, L. fermentum, L. brevis, Weissella soli*	Turkey, Asia, China
Cucumber	*P. pentosaceus, L. plantarum*, *L. brevis, Leuc. fallax*	USA, Europe, Asia, Turkey, China
Pepper	*Weissella confusa*, *Weissella cibaria*	Europe, Asia, USA
Red beet	*L. plantarum, L. pentosus*	Europe, Asia, USA

With the introduction of fermented vegetables in the human diet, this method of preservation of green fodder was applied in the nutrition of various species of animals. Traditionally, wet roughage is used for the production of silage administered to cattle. This method for preservation of fodder plants is also used in the nutrition of monogastric animals, e.g. poultry or pigs, which have a very sensitive gastrointestinal tract^4^. Due to the specificity of nutrient digestion and the physiological requirements of monogastric animals, feed has to be selected carefully in terms of both quality and quantity. No pre-fermentation occurs in the organism of these animals, and all activity of bacterial cultures takes place in the final segment of the gastrointestinal tract^5^. Fermented vegetables are often part of diets for these animals rather than forage used in cattle breeding^6^.

Modern science confirms that fermented products contain many health-enhancing components, e.g. organic acids, ethanol, or other antimicrobial compounds inhibiting the growth of microorganisms responsible for deterioration processes leading to food spoilage and growth of food-borne pathogens^7,8^. Moreover, fermentation ensures unique food flavors and aromas and improves food texture and appearance. Functionality and economic value are the other attributes of the fermentation process itself^9,10^.

Fermented food used in human nutrition or animal feed is currently an important element of the diet influencing the function of the organism and the gastrointestinal tract. Lactic acid bacteria are responsible for the beneficial effect on health and taste values of fermented products^11^. They improve the nutritional quality of food and control some processes in the organism, e.g. the synthesis of nutritionally important compounds and enhancement of the bioavailability of nutrients in fermented raw materials. They also synthesize cellular enzymes and other bioactive ingredients stabilizing the gastrointestinal tract environment and stimulate digestion processes^12,13^. The increase in the number of lactic acid bacteria (LAB) during fermentation enhances conversion of phenolic compounds (e.g. flavonoids) into biologically active metabolites through the expression of hydrolase, esterase, glycosyl decarboxylase, and phenolic acid reductase. The reaction of metabolites with anthocyanidins leads to the generation of e.g. alkyl catechols, which are capable of strong activation of oxidative stress response regulators in mammals. Thus, they induce the expression of antioxidant and detoxifying enzymes protecting the organism against oxidative stress and chemical damage^10,13^.

The fermentation processes are accompanied by multidirectional modification of the basic chemical composition of processed raw materials in terms of the content of mineral elements and vitamins. The type of compounds formed during fermentation depends on the type of microorganisms involved; for instance, vitamins B, including folates and B_12_, can be synthesized by plant-associated bacteria^11,14^. Fermentation may remove toxic or undesirable food components, such as phytic acid chelating divalent metal ions, or reduce the level of heavy metals.^15^

Therefore, the aim of the study was to analyze the impact of the lactic acid fermentation process on changes in the basic chemical composition, selected minerals, heavy metals, vitamins, and phenols. Additionally, the fermentation of broccoli, carrots, red peppers, and red beet was assessed in terms of pH and the presence of organic acids.

## Results

### Organic acids, pH, basic nutrients, and energy

The characteristics of the analyzed vegetables and the experimental design are shown in Table 2. Cucumber (CU.F) and peppers (PE.F) were the most easily fermented vegetables, which reached a stable fermentation level with an average pH value of 3.47 after 14 days (*P* = 0.027) (Table 3). The slowest fermentation rate was detected in broccoli, which was characterized by a pH value of 4.51 after 21 days. The intensity of fermentation was reflected in the level of lactic acid as well (*P* = 0.018). Its highest amount was determined in PE.F and BE.R (on average 33 g kg^−1^), and the lowest level was detected in BR.F and CA.F (on average 24.05 g kg^-1^). Significant differences were noted in the content of acetate acid (*P* = 0.031), which ranged from 3.8 to on average 4.4 g kg^−1^ (BR.F vs. CU.F, PE.F, and Be.F, respectively). The levels of propionate and butyrate were similar in all fermented vegetables.

**Table 2:** Scientific and common names of experimental vegetables and abbreviations used in the experimental scheme.

Vegetables	Scientific name	Source^a^	Abbreviations in the experimental scheme	
			Raw	Fermented
Broccoli	*Brassica oleracea var. italica*	Polish vegetable farm	BR.R	BR.F
Carrot	*Daucus carota*	Polish vegetable farm	CA.R	CA.F
Cucumber	*Cucumis*	Polish vegetable farm	CU.R	CU.F
Pepper	*Capsicum annuum*	Polish vegetable farm	PE.R	PE.F
Red beet	*Beta vulgaris subsp. vulgaris*	Polish vegetable farm	BE.R	BE.F

^a^According to the information from the seller.

**Table 3:** Content of organic acids (g kg^−1^) and pH of fermented vegetables.

Vegetables	Organic acids					pH			
	Lactic		Acetate	Propionate	Butyrate	7 day		14 day	21 day
BR.F	22.8 ± 0.24		5.3 ± 0.22	0.028 ± 0.26	0.008 ± 0.17	4.68 ± 0.28		4.66 ± 0.29	4.51 ± 0.45
CA.F	25.3 ± 0.15		3.8 ± 0.31	0.033 ± 0.45	0.005 ± 0.65	3.69 ± 0.15		3.58 ± 0.35	3.56 ± 0.41
CU.F	31.8 ± 0.62		4.2 ± 0.19	0.037 ± 0.18	0.009 ± 0.17	3.55 ± 0.34		3.49 ± 0.43	3.48 ± 0.37
PE.F	32.8 ± 0.18		4.5 ± 0.23	0.029 ± 0.21	0.006 ± 0.29	3.65 ± 0.25		3.47 ± 0.09	3.43 ± 0.44
BE.F	33.2 ± 0.35		4.6 ± 0.47	0.035 ± 0.34	0.007 ± 0.55	3.98 ± 0.19		3.65 ± 0.18	3.57 ± 0.31
ANOVA P-value^a^	0.018		0.031	0.125	0.245	0.039		0.027	0.022

Number of repetitions (n = 4).

^a^*P* < 0.05 statistical differences.

The analyzed vegetables also differed in their chemical composition and energy value (Table 4). The highest concentration of dry matter (*P* = 0.022) was detected in the raw BR.R, CA.R, and PE.R samples (18.71, 16.02, and 14.82 g 100 g^−1^), while approx. 3.4-fold lower dry matter content was determined in CU.R (5.98 g 100 g^−1^). In turn, the highest content of mineral elements in crude ash was recorded in CU.R and BE.R (on average 1.41 g 100 g^−1^). The highest amount of crude protein (*P* = 0.033) was found in BR.R and BE.R (on average 2.63 g 100 g^−1^) as well as CA.R and PE.R (on average 1.40 g 100 g^−1^). The highest levels of crude fiber (*P* = 0.013) were determined in the BR.R, CA.R, and PE.R samples (on average 3.79 g 100 g^−1^). CU.R had an approx. 25% lower amount of crude fiber compared with vegetables characterized by the highest amount of this nutrient. In most variants, the fermentation process induced loss of dry matter, crude ash, crude protein, and crude fiber (*P* < 0.019 MANOVA). An opposite phenomenon was observed in the case of ether extract and energy. Although the vegetables exhibited low levels of ether extract, which did not exceed 1% of the sum of all nutrients, an even two-fold increase in its content was noted in the fermented vegetables (CU.R-CU.F, PE.R-PE.F). This was also reflected in changes in the energy value of the vegetables (*P* = 0.027). The highest calorific value was calculated for BR.R, PE.R, and BE.R (on average 17.45 kcal in 100 g^−1^), whereas CU.R had the lowest value of the parameter (8.29 kcal in 100 g^−1^). The fermentation process also increased the calorific value of most of the tested vegetables, but the greatest differences, even by 34%, were found in the case of cucumber (CU.R-CU.F).

**Table 4:** Basic nutrients (g·100 g^−1^ fresh matter) and pH of raw and fermented vegetables.

Vegetables	Dry matter	Crude ash	Crude protein^a^	Ether extract	Fiber crude	Energy^b^ (kcal)
BR.R	18.71 ± 0.31	0.76 ± 0.18	2.83 ± 0.13	0.61 ± 0.35	3.67 ± 0.42	16.81 ± 0.51
BR.F	17.49 ± 0.47	0.84 ± 0.33	3.01 ± 0.45	0.84 ± 0.24	2.94 ± 0.21	19.60 ± 0.26
CA.R	16.02 ± 0.54	0.62 ± 0.42	1.32 ± 0.33	0.33 ± 0.16	3.73 ± 0.36	15.71 ± 0.21
CA.F	15.99 ± 0.32	0.73 ± 0.31	1.18 ± 0.41	0.35 ± 0.15	2.41 ± 0.35	12.69 ± 0.48
CU.R	5.98 ± 0.19	1.47 ± 0.23	0.95 ± 0.24	0.29 ± 0.52	0.94 ± 0.21	8.29 ± 0.16
CU.F	6.17 ± 0.42	1.62 ± 0.35	0.89 ± 0.37	0.68 ± 0.64	0.73 ± 0.53	11.14 ± 0.18
PE.R	14.82 ± 0.23	0.71 ± 0.56	1.49 ± 0.38	0.43 ± 0.29	3.98 ± 0.37	17.79 ± 0.41
PE.F	12.67 ± 0.35	0.83 ± 0.43	1.36 ± 0.56	0.91 ± 0.18	2.56 ± 0.44	18.75 ± 0.25
BE.R	13.53 ± 0.18	1.36 ± 0.21	2.43 ± 0.19	0.19 ± 0.16	3.16 ± 0.23	17.75 ± 0.51
BE.F	14.55 ± 0.24	1.43 ± 0.39	2.18 ± 0.31	0.23 ± 0.25	2.57 ± 0.11	15.93 ± 0.34
ANOVA *P*-value^c^	0.022	0.038	0.033	0.017	0.013	0.027
MANOVA^d^ *P*-value^c^			< 0.019			

Results are the average ± standard deviation of four repetitions (n = 4) of each vegetable combination.

^a^Calculated by Kjeldahl nitrogen N × 6.25.

^b^In 100 g fresh matter.

^c^*P* < 0.05 statistical differences.

^d^Raw/fermented vegetables.

### Macro-, microelements, vitamins, and phenols

The analyzed vegetables had different levels of macroelements (Table 5). Fresh carrots (CA.R) and red beet (BE.R) turned out to be the richest sources of Na (87.63 and 69.29 g kg^−1^ fresh matter, respectively). The smallest amounts of this microelement were detected in PE.R (9.47 g kg^−1^ fresh matter). The table salt (NaCl) additive used in the fermentation process significantly increased the Na content in the fermented vegetables (*P* = 0.018). The highest accumulation of Na in plant tissues, which was approx. fivefold higher than that in the raw vegetables, was recorded in vegetables that naturally have relatively low levels of the element (BR.F, CU.F, PE.F). The highest K content (*P* = 0.032) was determined in BR.R and BE.R (318.2 and 287.7 g kg^−1^ fresh matter, respectively), and the highest Ca level (*P* = 0.045) was detected in BR.R, CA.R, and BE.R (46.09, 32.64, and 36.04 g kg^−1^ fresh matter, respectively). The average magnesium level in a majority of the tested vegetables (BR.R, CA.R, PE.R, BE.R) was estimated at 21.5 g kg^−1^ fresh matter, while 60% lower content was detected in cucumber (*P* = 0.029). Large differentiation was also observed in the content of P in the vegetables (*P* = 0.037). Broccoli (BR.R, 69.07 g kg^−1^) as well as carrots and peppers (CA.R and PE.R—on average 38.86 g kg^−1^ fresh matter) turned out to be the richest sources of this mineral. The fermentation process reduced the content of K, Ca, Mg, and *P* in all variants (*P* < 0.013 MANOVA). In comparison with raw vegetables, the highest fermentation-induced losses were detected in the content of macronutrients: K in CU.F (25.5%), Ca in BE.F (32%), Mg in CU.F (24%), and P in PE.F and CU.R (40 and 28%, respectively).

**Table 5:** Macroelements in raw and fermented vegetables (g·kg^−1^ fresh matter).

Vegetables	Na	K	Ca	Mg	*P*
BR.R	18.47 ± 0.52	318.2 ± 0.29	46.09 ± 0.17	22.25 ± 0.37	69.07 ± 0.34
BR.F	85.22 ± 0.31	285.4 ± 0.53	42.12 ± 0.42	18.34 ± 0.29	61.12 ± 0.57
CA.R	87.63 ± 0.43	105.1 ± 0.34	32.64 ± 0.39	22.62 ± 0.37	39.31 ± 0.24
CA.F	164.8 ± 0.29	96.09 ± 0.33	28.13 ± 0.34	19.06 ± 0.46	33.48 ± 0.37
CU.R	15.37 ± 0.16	132.5 ± 0.38	18.82 ± 0.19	11.32 ± 0.41	24.54 ± 0.29
CU.F	81.3 ± 0.35	98.32 ± 0.17	20.32 ± 0.37	8.64 ± 0.25	17.63 ± 0.44
PE.R	9.47 ± 0.21	187.2 ± 0.53	17.23 ± 0.46	21.61 ± 0.31	38.41 ± 0.39
PE.F	44.5 ± 0.37	169.1 ± 0.44	15.45 ± 0.19	19.22 ± 0.28	23.15 ± 0.26
BE.R	69.29 ± 0.19	287.7 ± 0.28	36.04 ± 0.27	19.57 ± 0.36	23.09 ± 0.14
BE.F	114.4 ± 0.21	226.2 ± 0.19	24.38 ± 0.34	16.37 ± 0.52	19.11 ± 0.19
ANOVA *P*-value^a^	0.018	0.032	0.045	0.029	0.037
MANOVA^b^ *P*-value^a^			< 0.013		

The results are the mean ± standard deviation of four repetitions (n = 4) of each vegetable combination.

^a^*P* < 0.05 statistical differences.

^b^Raw/fermented vegetables.

In the group of the analyzed microelements, the highest content of Zn (*P* = 0.023) and Fe (*P* = 0.045) was exhibited by broccoli and red beet (BR.R, BE.R: on average 0.31 and 0.84 mg 100 g^−1^ fresh matter, respectively). The highest Cu level was determined in cucumber and red beet (CU.R, BE.R: on average 0.09 mg 100 g^−1^ fresh matter) (Table 6). In all fermented vegetables, the content of the analyzed microelements declined in comparison with the raw material (*P* < 0.07 MANOVA). The highest losses in the Zn content, amounting even to 47%, were recorded during the fermentation of broccoli (BR.F) and red beet (BE.F). The BE.F samples exhibited the highest (approx. 32%) reduction in the Cu content, compared with the raw vegetables. In turn, in terms of the Fe content, cucumbers (CU.F) and peppers (PE.F) turned out to be the most susceptible to the fermentation process, as an average 23% loss of this microelement was noted in the fermented samples of these vegetables. The analyzed raw vegetables contained from 8.28 (CA.R) to 4.28 µg g^−1^ fresh matter (PE.R) of Pb (*P* = 0.036) and from 0.057 (CU.R) to 0.031 µg g^−1^ fresh matter (BE.R) of Cd (*P* = 0.027). As in the case of the other microelements, a decline in the Pb content was recorded during the fermentation process. The loss was most intense in the CA.F, CU.F, and BE.F samples. In these vegetables, the content of this heavy metal was on average 18.5% lower than in the raw material. Slightly lower reduction of the contamination was noted in the case of the Cd content. The highest decline, on average by 12% vs. its level in the raw vegetables, was recorded in CA.F, PE.F, and BE.F.

**Table 6:** Microelements and heavy metals in raw and fermented vegetables.

Vegetables	Zn^a^	Cu^a^	Fe^a^	Pb^b^	Cd^b^
BR.R	0.29 ± 0.17	0.034 ± 0.34	0.85 ± 0.39	4.39 ± 0.71	0.041 ± 0.37
BR.F	0.15 ± 0.28	0.023 ± 0.46	0.77 ± 0.26	3.78 ± 0.23	0.039 ± 0.64
CA.R	0.25 ± 0.37	0.046 ± 0.27	0.35 ± 0.19	8.28 ± 0.51	0.043 ± 0.26
CA.F	0.19 ± 0.19	0.039 ± 0.29	0.28 ± 0.44	6.73 ± 0.34	0.038 ± 0.44
CU.R	0.21 ± 0.53	0.094 ± 0.16	0.26 ± 0.21	5.30 ± 0.45	0.057 ± 0.53
CU.F	0.16 ± 0.25	0.086 ± 0.34	0.19 ± 0.53	4.32 ± 0.18	0.054 ± 0.24
PE.R	0.23 ± 0.34	0.059 ± 0.19	0.39 ± 0.37	4.28 ± 0.23	0.026 ± 0.19
PE.F	0.21 ± 0.28	0.053 ± 0.22	0.35 ± 0.42	3.97 ± 0.25	0.023 ± 0.37
BE.R	0.32 ± 0.19	0.086 ± 0.36	0.83 ± 0.56	5.02 ± 0.39	0.031 ± 0.26
BE.F	0.17 ± 0.56	0.079 ± 0.16	0.76 ± 0.14	4.12 ± 0.24	0.027 ± 0.51
ANOVA *P*-value^c^	0.023	0.045	0.018	0.036	0.027
MANOVA ^d^ *P*-value^c^	< 0.007				

The results are the mean ± standard deviation of four repetitions (n = 4) of each vegetable combination.

^a^mg·100 g^−1^ fresh matter.

^b^µg g^−1^ fresh matter.

^c^*P* < 0.05 statistical differences.

^d^Raw/fermented vegetables.

The vegetables selected for the study were also characterized by high variability in the content of vitamins and phenols (Table 7). The highest levels of vitamin C (*P* = 0.037) were determined in the PE.R and BR.R samples (138.6 and 95.25 mg 100 g^−1^ fresh matter, respectively), whereas its average level in CA.R and BE.R was 4.9 mg 100 g^−1^ fresh matter. The highest content of vitamin A (*P* = 0.025) and carotene (*P* = 0.019) was determined in CA.R and in PE.R and BR.R (in the range from 987.5 and 1109 to 257.5 and 738.4 µg g^−1^ fresh matter). In turn, these compounds in the red beet were present only at the level of 2.35 (vitamin A) and 18.56 µg g^−1^ fresh matter (carotene). High variability was also noted in the content of total phenols (*P* = 0.041), which were present on average in the range from 787 (BR.R and PE.R) to even 25.9 µg g^-1^ fresh matter (CU.R). Similar to minerals, significant losses induced by the fermentation process were noted in the content of vitamins and phenols (*P* < 0.012 MANOVA). The highest losses, i.e. on average by 50%, in the content of vitamin C were recorded in vegetables that contained the lowest levels of this component (CA.F, BE.F, CU.F). Slightly different changes were observed in the case of vitamin A and carotenes. In some of the fermented vegetables, the content of these compounds decreased in comparison with the unprocessed raw material. The maximum loss of these bioactive compounds induced by the fermentation process amounted to 22% of vitamin A (BR.F and CU.F) and 25% of carotenes (CU.F). In turn, the fermented carrots and peppers had a 34–23% higher level of vitamin A (CA.F–PE.F) and approx. 24% higher content of carotenes (CA.F). A decrease in the content of total phenols in the vegetables was induced by the fermentation process. The highest losses of these compounds, i.e. 33 and 26%, were exhibited by CU.F and PE.F, respectively, compared to the raw material, whereas on average 21% losses of these bioactive compounds were recorded in the other fermented vegetables.

**Table 7:** Chosen vitamins and phenols in raw and fermented vegetables.

Vegetables	Vitamin C^a^	Vitamin A^b^	Carotene^b^	Phenols (total)^b^
BR.R	95.25 ± 0.34	126.2 ± 0.29	386.2 ± 0.33	876.4 ± 0.21
BR.F	78.32 ± 0.25	98.65 ± 0.41	347.5 ± 0.25	718.3 ± 0.33
CA.R	4.89 ± 0.41	736.1 ± 0.37	895.3 ± 0.39	476.5 ± 0.58
CA.F	2.16 ± 0.15	987.5 ± 0.61	1109 ± 0.41	365.9 ± 0.24
CU.R	5.73 ± 0.33	19.54 ± 0.19	49.62 ± 0.32	25.9 ± 0.41
CU.F	3.28 ± 0.48	15.39 ± 0.22	37.18 ± 0.56	17.4 ± 0.32
PE.R	138.6 ± 0.32	208.6 ± 0.39	695.9 ± 0.27	698.2 ± 0.28
PE.F	119.2 ± 0.18	257.5 ± 0.26	738.4 ± 0.42	516.3 ± 0.36
BE.R	4.92 ± 0.34	2.35 ± 0.45	18.56 ± 0.53	221.5 ± 0.32
BE.F	2.34 ± 0.51	2.19 ± 0.26	15.73 ± 0.40	174.6 ± 0.19
ANOVA *P*-value^c^	0.037	0.025	0.019	0.041
MANOVA^d^ *P*-value^c^		< 0.012		

The results are the mean ± standard deviation of four repetitions (n = 4) of each vegetable combination.

^a^mg·100 g^-1^ fresh matter.

^b^µg g^-1^ fresh matter.

^c^*P* < 0.05 statistical differences.

^d^Raw/fermented vegetables.

### Calculation of dietary intake via consumption of one serving of raw and fermented vegetables

#### Humans

The results demonstrated that the consumption of one serving of fresh analyzed vegetables provided basic nutrients in the range of approx. 1.04–3.11% of daily protein (CU.R–BR.R), 0.29–1.40% of daily fat (BE.R – PE.R), and 0.41- average 0.86% of daily energy (CU.R–BR.R and BE.R) (Table 8). In a majority of the analyzed vegetables, the fermentation process reduced the amount of the basic nutrients that could potentially be used in the daily ration of human nutrition.

**Table 8:** Percent coverage of the daily supply of selected nutrients, minerals, and vitamins via human consumption of one serving of raw and fermented vegetables.

Vegetables	Protein	Fat	Energy	Ca	Mg	Zn	Cu	Fe	Vitamin C	Vitamin A
BR.R	3.11	0.94	0.84	576	530	2.64	1.42	7.73	106	14.0
BR.F	3.31	1.29	0.98	527	437	1.36	0.96	7.00	87.0	10.9
CA.R	1.45	0.51	0.79	408	539	2.27	1.92	3.18	5.43	81.8
CA.F	1.30	0.54	0.63	352	454	1.73	1.63	2.55	2.40	110
CU.R	1.04	0.45	0.41	235	270	1.91	3.92	2.36	6.37	2.17
CU.F	0.98	1.05	0.56	254	206	1.45	3.58	1.73	3.64	1.71
PE.R	1.64	0.66	0.89	215	515	2.09	2.46	3.55	154	23.2
PE.F	1.49	1.40	0.94	193	458	1.91	2.21	3.18	132	28.6
BE.R	2.67	0.29	0.89	451	466	2.91	3.58	7.55	5.47	0.26
BE.F	2.40	0.35	0.79	305	390	1.55	3.88	6.91	2.60	0.24
Daily intake^a,b^	91 g	65 g	2000 kcal	0.8 g	0.3–0.42	6–11 mg	0.9–2.4 mg	8–11 mg	75–90 mg	700–900 µm

One serving: 100 g accepted as an average portion^17^.

^a^Dietary Guidelines for Americans^17^.

^b^Food Standards for the Polish Population^18^.

Vegetables are a rich source of minerals and vitamins in the daily human diet (Table 8). The raw vegetables analyzed in the present study covered the required or recommended levels for minerals in human nutrition as follows: 193–576% of Ca (PE. – BR.R), 206–530% of Mg (CU.R – BR.R), 1.73–2.91% of Zn (CA.R – BE.R), 1.42–3.92% of Cu (BR.R–CU.R), 2.36–7.73 of Fe (CU.R–BR.R), 5.34–154% of vitamin C (CA.R and BE.R – PE.R), and 0.26–110% of vitamin A (BE.R–CA.R). As in the case of the basic nutrients, the amounts of available minerals and vitamins C and A decreased in the fermentation process in a majority of the vegetables.

#### Animals

Vegetables are a source of nutrients in animal nutrition as well (Table 9). The amount of 100 g of dry matter of vegetables in the feed mixture can provide the organism of ducks with approx. 0.28–1.64% of daily protein (CU.R–BR.R), 0.11–1.06% of ether extract (BR.R–PE.R), 0.72–8.54 of crude fiber (BR.R–CA.R), and 0.27- average 0.55% of daily energy (CU.R – BR.R, CA.R, PE.R and BE.R). The values in the case of pigs are as follows: approx. 0.04–0.37% of daily protein (CU.R–BR.R), 0.19- average 1.27% of ether extract (CU.R–BR.R, PE.R), 0.62–6.64 of crude fiber (CU.R–CA.R), and 0.42- average 0.57% of daily energy (CU.R–BR.R, CA.R, PE.R, and BE.R). The fermentation process reduced the amount of vegetable-derived crude protein and fiber available for the monogastric animals and increased the amount of ether extract and energy.

**Table 9:** Percent coverage of daily demand for selected nutrients, minerals, and vitamin A by raw and fermented vegetables (100 g dry matter) consumed by monogastric animals (ducks and pigs).

Vegetables	Crude protein	Ether extract	Crude fiber	Energy	Na	Ca	Mg	*P*	Zn	Vitamin A
**Ducks**^**a**^										
BR.R	0.53	0.11	0.72	0.54	15.19	5.86	5.18	21.46	0.068	4.70
BR.F	0.52	0.15	0.52	0.63	64.27	4.91	3.92	17.41	0.032	3.37
CA.R	1.06	0.88	8.54	0.51	82.58	4.75	6.04	13.99	0.067	31.39
CA.F	0.94	0.93	5.51	0.41	155	4.09	5.08	11.90	0.051	42.19
CU.R	0.28	0.29	0.80	0.27	5.41	1.02	1.13	3.26	0.021	0.31
CU.F	0.27	0.70	0.64	0.36	29.51	1.14	0.89	2.42	0.016	0.25
PE.R	1.10	1.06	8.43	0.57	8.26	2.32	5.34	12.65	0.057	7.05
PE.F	0.86	1.92	4.63	0.60	33.17	1.78	4.06	6.52	0.044	10.18
BE.R	1.64	0.43	6.11	0.57	55.15	4.43	4.41	6.94	0.072	0.08
BE.F	1.59	0.56	5.34	0.51	97.91	3.22	3.97	6.18	0.041	0.09
**Pigs**^**b**^										
BR.R	0.37	1.27	8.25	0.55	2.35	1.34	2.59	2.41	0.051	8.82
BR.F	0.38	1.63	5.71	0.65	9.93	1.12	1.96	2.28	0.024	6.32
CA.R	0.15	0.59	6.64	0.53	12.76	1.09	3.02	1.96	0.050	79.10
CA.F	0.13	0.62	4.28	0.42	23.96	0.94	2.54	1.34	0.038	58.85
CU.R	0.04	0.19	0.62	0.28	0.84	0.23	0.56	0.37	0.016	0.58
CU.F	0.03	0.47	0.50	0.37	4.56	0.26	0.44	0.27	0.012	0.47
PE.R	0.16	0.71	6.55	0.60	1.28	0.53	2.67	1.42	0.043	19.08
PE.F	0.12	1.28	3.60	0.63	5.13	0.41	2.03	0.73	0.033	13.21
BE.R	0.23	0.29	4.75	0.59	8.52	1.02	2.21	0.78	0.054	0.16
BE.F	0.24	0.37	4.15	0.53	15.13	0.74	1.98	0.70	0.031	0.15

^a^Coverage for broiler ducks in rearing period III (finisher) in 1 kg of feed^19^.

^b^Coverage for fatteners in rearing period III (finisher) in 1 kg of feed^20^.

In animal nutrition, vegetables are an important source of macro- and microelements as well as vitamins, regardless of supplementation with synthetic preparations (Table 9). The analyzed vegetables provided significant amounts covering the daily requirement in the case of the following minerals: Na: 5.41–82.54% (CU.R – CA.R) and 0.84–12.76% (CU.R–CA.R) (ducks and pigs, respectively), P: 3.26–21.46% (CU.R – BR.R, ducks), Ca: 1.02–5.86% (CU.R – BR.R, ducks), Mg: 1.13–6.04 and 0.56–2.41% (CU.R – BR.R, ducks and pigs). In the case of Ca and Zn daily requirement, the levels of Ca did not exceed 1.5% in pig nutrition and 0.08% of Zn daily demand in the nutrition for ducks and pigs. The content of vitamin A in the analyzed vegetables covered the daily demand in the range of 0.31–42.19 and 0.58–79.10% (ducks and pigs, CU.R–CA.R, respectively). The addition of salt as a preservative during the fermentation process caused a several-fold increase in the Na content in the fermented vegetables. In turn, the content of other minerals and vitamin A in the fermented vegetables covered a smaller percentage of the daily requirement for these nutrients than in the raw vegetables. The largest differences in the potential to cover the nutritional demand by the raw and fermented vegetables were noted in the case of Zn (even by over 50% BR.F) and vitamin A (on average by 30% BR.F, PE.F).

## Discussion

Lactic fermentation is a natural process carried out by lactic acid bacteria (LAB) representing the orders *Lactobacillales*, *Bacillales*, and *Bifidobacteriales*^8^. They metabolize carbohydrates in biochemical processes, with lactic acid as the main metabolism product. It is a dominant organic acid produced by microorganisms during fermentation. In the analyzed vegetables subjected to the fermentation process, lactic acid constituted from 87 (cucumber) to 81% (broccoli) of the entire pool of organic acids. A similar composition of organic acids was also reported by Liu et al.^21^ in investigations of fermented broccoli leaves and stalks, where the microbiota was dominated by lactic acid bacteria. Similarly, in their study of some traditionally fermented vegetables, i.e. cucumber, carrots, olives, and cabbage, Dallal et al.^22^ found that lactic acid bacteria were the main microorganisms responsible for the fermentation process. However, the type of fermentation bacteria depends on the type of vegetables, geographic location, temperature, and even harvesting conditions and preparation method^23^. During fermentation, in addition to organic acids (lactic and acetic), lactic acid bacteria (LAB) produce other substances, e.g. diacetyl, ethanol, hydrogen peroxide, reuterin, acetaldehyde, acetoine, carbon dioxide, and bacteriocins^24^. These natural compounds serve as biopreservative agents inhibiting the growth of pathogenic, nonpathogenic, and spoilage microorganisms. They increase the safety of fermented food, thus extending the shelf life of food products^25^. Moreover, these ingredients have a beneficial effect by improving food flavor, aroma, and texture. Spontaneous fermentation of food ensures greater intensity of flavor and aroma than a controlled process based on the use of pure bacterial cultures^7,26^. In addition to the presence of biologically active substances generated during the fermentation process, the safety of fermented food is undoubtedly ensured by the reduced pH value, which stimulates the growth of fermentation bacteria in the range of 3.5–4.5, effectively inhibiting the multiplication of pathogenic microorganisms in food. The degree and rate of progressive acidification of plant material depends on its type and susceptibility to the fermentation process. A pH value of approx. 3.96 in traditional fermented Iranian vegetables was reported by Dallal et al.^22^, and pH in the range of 4.4–4.7 was determined in kimchi by Choi et al.^9^. In turn, the pH of the fermented Polish vegetables analyzed in the present study ranged on average from 3.51 to 4.51 (carrots, cucumber, red peppers, red beet, and broccoli).

The content of basic nutrients determined in the analyzed vegetables was similar to values reported by other researchers^27–32^. The fermentation process reduced most of the basic nutrients in the vegetables. Reduced crude ash, crude protein, and fiber contents in fermented corn seeds were observed by Ejigui et al.^1^. A similar phenomenon was reported by Ifesan et al.^33^ in their investigations of leafy vegetables *Amaranthus hybridus*, *Telfairia occidentalis*, *Vernonia amygdalina*, and *Pterocarpus mildbraedii* fermented in a traditional way. Losses of nutrients during the fermentation process are associated with the increasing nutritional needs of the growing lactic acid bacteria and meeting their metabolic requirements when the fermented plant material becomes a medium for these microorganisms^22^. Researchers highlight the role of the degree of hydration of raw material subjected to fermentation, e.g. by repeated rinsing, soaking, and addition of water, as it can largely determine the intensity of changes in the content of basic nutrients in fermented plants. A relatively low water level in relation to the dry matter of fermenting plant material may increase acidity but concurrently inhibit the development of the desired microbiota^34^. However, despite the lower content of protein in fermented foods, a higher degree of digestibility of this nutrient has been reported^35^. During fermentation, proteases produced by microorganisms partially degrade and release some proteins, thereby increasing the levels of peptides and free amino acids. Higher bioavailability has been determined in the case of cystine, histidine, and asparagine. Additionally, reduced amounts of anti-nutritional compounds promoting protein cross-linking (e.g. phenolic and tannin compounds) and inhibiting digestive enzymes (e.g. trypsin and chymotrypsin inhibitors) have been reported^36^.

The present study has shown an increase in the fat content in the fermented vegetables, especially in the broccoli, cucumber, and peppers. A similar effect of fermentation was reported by Ifesan et al.^33^ in analyses of green parts of grapevine and amaranth, where the increase in the content of this nutrient reached from 20 to even 40%. Nevertheless, contradictory data can be found in the literature as well. In a study conducted by Aziz et al.^37^, the reduction of the content of fat and energy in fermented broccoli and artichoke was ascribed to a decline in the carbohydrate fraction. The authors emphasize that the value of metabolic energy and other energy values are largely influenced by e.g. crude fiber levels during the vegetation period. In addition to the type of plant material, soil fertility, climatic conditions, and agrotechnical measures have an impact on changes in nutrient levels during the fermentation process^38^.

The mineral composition of the vegetables selected for the present study confirms that they can be a good source of minerals in the diet of humans^10,33^ and animals^39^. Very high levels of Na were determined in the fermented vegetables, which was associated with the use of salt in the traditional fermentation process. Such high salt content in fermented vegetables constitutes a certain limitation to the consumption thereof by humans^40^ and animals^41^. On the other hand, salt enhances the palatability of meals in the human diet^42^ and animal feed mixtures, where it is sometimes added to mask the flavor of feed materials with lower sensory attractiveness^43^. Besides its role in the sensory value, a high concentration of Na^+^ enhances the transport of active and nutritional substances into the bacterial cell, thereby accelerating and optimizing the fermentation process^44^. The levels of most macroelements were lower in the fermented vegetables than in the raw material. The fermentation process is accompanied with leakage of sap together with certain amounts of minerals from plant tissues^45^. However, minerals, especially Fe^2+^, Ca^2+^, Na^+^, Mg^2+^, and Zn^2+^, are indispensable for a proper course of the fermentation process^46^. In particular, Fe^2+^ and Ca^2+^ ions serve as coagulants necessary for agglomeration and multiplication of anaerobic microorganisms during the fermentation process. Moreover, Ca^2+^ ions are biocatalytic ions for microorganisms accelerating the biodegradation process. However, an excess concentration of these ions may inhibit the growth of lactic acid bacteria via dehydration of their cells. Magnesium ions in fermented vegetables act as mediators and support substrate utilization for optimization of the bacterial metabolic pathway^47^. The fermentation of the vegetables contributed to a favorable phenomenon, i.e. reduction of the level of such heavy metals as Cu, Pb, and Cd, which are relatively difficult to remove from plant material via culinary processing or during the preparation of animal feed. This phenomenon is probably the result of metal binding to the cell walls of fermentation bacteria and fungi. These metals are captured mainly in the adsorption process (ionic, chemical, and physical). The carboxyl, amino, hydroxyl, phosphate, and sulfhydryl groups present in the external structures of microorganisms are involved in heavy metal chelation^48^. Most frequently, heavy metal ions are adsorbed via complexation with negatively charged reaction sites on the cell surface^49^. As reported by Salehizadeh and Shojaosadati^50^, bacteria of the genus *Bacillus* are effective in binding Pb, Zn, and Cu. Microorganisms fermenting various parts of green plants may exhibit varied efficiency in removal of heavy metals. The highest effectiveness in the removal in the order Ni > Cd > Pb > Cu > Cr was exhibited by fermented root biomass roots. In turn, stem and leaf biomass removed metals in the following order: Ni > Pb > Cd > Cr and Ni > Cd > Cu > Pb > Cr, respectively^15^. As shown by Joshi et al.^51^, carrot pulp fermenting bacteria were very effective in binding Pb, Ni, Zn, and Fe.

Fermentation of vegetables is often associated with changes in the content of vitamins and other biologically active substances. Vitamins are essential to human and animal health, as these molecules are co-factors for many enzymes involved in all types of metabolism supporting the function of organs^52^. The vitamin C content in the analyzed material was reduced by up to 50%. A similar phenomenon and magnitude thereof were reported by Ifesan et al.^33^ in studies of grapevine leaves, *Pterocarpus mildbraedii*, and *Vernonia amygdalina* and by Grzelakowska et al.^14^, who analyzed cucumbers. With its strong antioxidant properties, vitamin C is a very good preservative of fermented vegetables. It plays an important role in the regulation of antioxidant mechanisms protecting cells and body fluids against oxidative stress (i.e. it affects the rate of aging). However, vitamin C is one of the most unstable vitamins. It is easily decomposed by under ultraviolet radiation, elevated temperature, and heavy metals. As suggested by Grzelakowska et al.^14^, fermentation-related vitamin C losses are determined by two factors: the release and activation of ascorbinase from damaged cell structures and the oxygen access. The low pH values noted during fermentation may limit the action of ascorbinase; hence, the decrease in the vitamin C content is mainly related to the process of oxidation with atmospheric oxygen^14^. In the fermented vegetables analyzed in the present study, the content of vitamin A and carotenes declined in comparison with their levels in the raw material. Carotenes are bioactive substances serving e.g. as vitamin A precursors. Researchers have reported variable effects of fermentation of vegetables on the level of these compounds^13,53^. They emphasize that changes in the β-carotene content during fermentation are highly variable, depending on the plant material and fermentation conditions. Similarly, both losses (broccoli, cucumber, red beet) and an increase in their content (carrots, red peppers) were recorded in the present study. An increase in carotene content in fermented peppers was reported by Lee et al.^53^. Other changes observed in the composition of these compounds included an increase in the content of volatile carotenoid derivatives, e.g. β-ionone, β-cyclocitral, α-ionone, and β-damascenone, which gradually increased during the fermentation process. β-damascenon is characterized by fruity and floral aromas, which significantly improves the flavor and aroma of fermented vegetables. An almost 70% increase in the carotene content in fermented tomatoes was reported by Bartkiene et al.^31^. As shown by Chavasit et al.^54^, bacteria occurring naturally in fermented vegetables, e.g. Chinese cabbage, have an ability to induce an apparent increase in the content of β-carotene after 2 weeks of storage. The authors noted a visible increase in the content of β-carotene ranging from 30 to 40%. They explained these results by the enhanced release of β-carotene from carrots during processing and even storage. It is assumed that the increase in carotenes in fermented plants may actually be related to structural changes induced by fermentation, which may increase the ability to extract carotenoids^13^. There are also many reports of carotene losses in the process of fermentation of vegetables. Such a phenomenon was reported by Kun et al.^55^ after 24-h fermentation of carrot juice by Bifidobacteria. The authors found that the levels of α-carotene and β-carotene were reduced by even 25%. Similar carotene losses in fermented tomatoes accompanied by a substantial increase in the level and bioavailability of lycopene were found by Cooperstone et al.^56^. Slightly different conclusions were formulated by Oloo et al.^12^ in their investigations of sweet potatoes and oranges fermented by *Lactobacillus plantarum*. They recorded approximately 94% β-carotene retention after the fermentation process. As emphasized by the authors, lactic acid fermentation preserves β-carotene much more efficiently than other technologies of vegetable food and feed processing, e.g. steaming, blanching, or drying.

Different levels of antioxidant activity are observed in plant-derived foods subjected to lactic acid fermentation, which is probably related to the release of bioactive compounds with such conjugate phytochemicals as phenols. Nevertheless, the phenolic compound metabolism by lactobacilli in food fermentation is still poorly characterized^57^. The levels of these compounds in fermented vegetables are most often reduced, which is also accompanied by a decline in the content of flavonoids. Concurrently, the activity of lycopene and antioxidants is enhanced^58^. An opposite phenomenon was noted in studies of fruits. Sirilun et al.^59^ reported an increase in the total phenolic content and the antioxidant activity in *Syzygium cumini* L. fruit juice fermented by *Lb. paracasei* strain HII01, and Bujna et al.^60^ observed an increase in the amount of phenols and antioxidant activity in apricot juice. The functional value of vegetable phenols is well known, but their biological properties depend on the degree of absorption thereof in the gastrointestinal tract. Human and animal tissues and biological fluids do not contain esterases, which hydrolyze chlorogenic acid in order to release caffeic acid. This task is carried out only by the gastrointestinal microbiota. Free caffeic acid is absorbed in the stomach and small intestine more efficiently than chlorogenic acid^61^. Dihydrocaffeic acid exhibits high bioavailability and better antioxidant properties than its precursor, i.e. caffeic acid^62^. Therefore, through bacterial bioconversion, fermented vegetables are enriched with phenolic derivatives with increased bioavailability to humans and animals. As shown by Filannino et al.^57^ in investigations of fermented broccoli puree, *Lactobacillus* spp. strains exhibited specific metabolism of phenolic acids, e.g. hydroxybenzoic acids (protocatechuic acid), hydroxycinnamic acids (caffeic and p-coumaric acids), and hydroxycinnamic acid derivatives (chlorogenic acid). Similarly, *L. plantarum* consistently contributed to the highest functional value of modulation of phenol metabolism in fermented vegetables. The fermentation process carried out in this way yielded a product with an above-average content of phenolic acids that were highly bioavailable to the human or animal organism.

Vegetables provide nutrients necessary for the health and proper function of the organism. They are naturally low in fat and calories. In human and animal diets, they are the main source of bioactive substances, e.g. minerals, vitamins, or other biologically active compounds^11^. Vegetables are recommended as the basis of the diet in human nutrition. For the adult population, 4–5 daily servings of vegetables are recommended for consumption at regular intervals^18^. Although it reduced the content of most basic nutrients, minerals, and vitamins, the fermentation process is regarded by nutritionists as beneficial to human health. It contributes to an increase in the bioavailability of these nutrients and stabilization of the gastrointestinal tract environment due to the presence of lactic acid bacteria and reduced pH values^24,61^. Additionally, the probiotic bacteria present in fermented food can boost metabolism and intensify decomposition and removal of toxins. Subjects that consume greater amounts of products from this group as part of a healthy balanced diet are less likely to develop some chronic diseases, e.g. obesity or gastrointestinal diseases. Additionally, their immune system is mobilized to ensure higher resistance to viral, bacterial, and fungal infections^63^. A risky component of traditionally fermented food is the high salt content, especially in pickled cucumbers; hence, it is recommended to limit consumption of these products^64^.

As in the human diet, the presence of fermented plants is recommended in animal nutrition. The fermentation process modifies the chemical composition of preserved vegetables, e.g. it reduces the concentration of dry matter and certain nutrients. The reduction of the content of dietary fiber, i.e. one of the most important nutrients in feed for animals, is a favorable phenomenon in poultry and pig nutrition^65,66^. Although dietary fiber should be contained in feed rations at the levels of 3–4% in piglets, 6% in fattening pigs, 10% in sows, 3–4% in broiler chickens and turkeys, 5–9% in geese, and 5–6% in ducks for proper functioning of the gastrointestinal tract, its excess may deteriorate the digestibility of feed mixtures^19,20^. Fiber enhances intestinal peristalsis and shortens the food transit time in the intestines. Its excess amounts in the food ration may also cause deficiencies in energy, protein, or other nutrients. It can also cause flatulence and diarrhea, which only exacerbates the problem of worsened feed conversion and a decline in weight gain rates^67,68^. Currently, the nutrition of animals in intensive production is based on industrial fodder, which mainly consists of dietary fiber-rich cereals. The introduction of fermented fodder into animal diet can optimize the amount and quality of fiber. The safety of administration of silage to animals is ensured by its high quality achieved by fermentation of high-quality vegetables and maintenance of anaerobic conditions with rapid achievement of low pH^69^. Chemical and biological additives that rapidly reduce silage pH and prevent oxygen degradation are frequently used in animal production^70^. Properly prepared and administered silage is an excellent feed with considerable health-enhancing and probiotic effects on farm animals^71^. However, various undesirable microorganisms can colonize silage at an insufficiently low pH value or oxygen availability. A higher probability of such spoilage is noted when large amounts of plants for animals are ensiled in piles or bales^72^. The undesirable microorganisms are represented by e.g. *Clostridium botulinum*, *Bacillus cereus*, *Listeria monocytogenes*, *Escherichia coli*, other *Enterobacteriaceae* species, and molds. Animal health may also be threatened by metabolites produced by these microorganisms, e.g. butyric acid or mycotoxins produced by certain molds^6^. Therefore, to ensure the safety of silage, it is important to monitor its quality constantly at every stage of preparation and application.

## Conclusion

Both fresh and fermented vegetables are good sources of nutrients and bioactive compounds. The largest amounts of basic components were recorded for the concentration of dry matter (broccoli and carrots) and crude fiber (broccoli, carrots, peppers, and red beet). The highest levels of Ca and Mg were found in broccoli, carrots, and red beet, K in broccoli and red beet, and vitamin A, carotene, and phenolic compounds in carrots, peppers, and broccoli. The fermentation process reduced the water content in plant tissues, which contributed to a relative increase in the concentration of nutrients, with the exception of crude fat and energy. The fermentation process contributed to the generation of large amounts of lactic acid in the vegetables, which can stabilize the gastrointestinal tract environment. Additionally, the process increased the amounts of vitamin A and carotene in carrots and red peppers, while reduced levels of these components were observed in the other vegetables. All fermented vegetables were characterized by lower levels of heavy metals, i.e. Cu, Pb, and Cd. Despite the relative loss of some nutrients and bioactive compounds, the physical and chemical changes induced by the fermentation process provided vegetables with new functionalities, e.g. probiotic character and durability. The reduced pH of the vegetables increased their microbiological stability and extended their shelf life.

## Materials and methods

### Plant material and fermentation conditions

The investigations were conducted on five vegetables species. All methods were used in accordance with the relevant UE guidelines/regulations/legislation. Vegetables that are most often fermented in traditional cuisines and used in animal nutrition in Europe (including Poland), Asia, and America were selected for the investigations (Table 1). The characteristics of the analyzed vegetables and the experimental design are shown in Table 2. Whole fresh plant materials were purchased in a specialist shop in 2019 (Lublin, Poland). As specified by the seller, the raw vegetables originated from a farm localized in the southeast of Poland.

The fermentation process was carried out in accordance with the traditional recipe used in the Polish cuisine. 1-L sterile glass jars were used for the fermentation. Approximately 600 g of raw vegetables were shredded into ca. 1 cm thick pieces and placed in the jars. Spice additives that are used traditionally in Eastern Europe for vegetable fermentation, i.e. 20 g of horseradish (*Armoracia rusticana*), 10 g of garlic (*Allium sativum*), 0.2 g of pepper (*Piper nigrum*), 0.5 g of white mustard (*Sinapis alba*), and 20 g of dill (*Anethum graveolens*), were placed in each jar. The vessels with the material were filled with brine with a NaCl concentration of 2.5%. The amount of the brine was sufficient to cover the sliced vegetables completely. The jars were then covered with screw caps and kept at 24 °C for 3 weeks. Afterwards, the jars were transferred to refrigeration conditions (6 °C) in order to slow down and equalize the fermentation activity. Four repetitions of the fermentation process with four jars containing fermented vegetables were carried out (for each vegetable separately).

### Organic acids, pH measurement, and basic composition

The content of organic acids in the fermented vegetables was determined with gas chromatography on an Agilent Technologies 6890 N gas chromatograph with a FID detector; carrier: helium; HP-5 capillary column with an internal diameter of 320 μm and a length of 28.5 m; FID detector temperature 250–260 °C, 2.0 min^73^.

The proper course of the fermentation process was verified via weekly measurements of the acidity of the fermentation juice. The optimal acidity of pH 3–4 for lactic acid bacteria inhibiting the development of potential pathogens was assumed as the fermentation safety criterion, as proposed by Montet et al.^16^. The measurements were carried out using a Pen Type Meters EZDO 6011A Waterproof pH Meter with a regular electrode (GMM, Taiwan).

The chemical analyses were conducted in three replications of each combination of vegetables. The content of dry matter (Method 44-15A) and basic nutrients (crude ash—Method 08-01, crude protein—Method 46-06, ether extract (crude fat determined with the Soxhlet method)—Method 30-10) in the vegetables samples (250 g of each vegetables) was determined according to standard AOAC^74^ procedures.

The energy value of the analyzed vegetables was based on the Atwater general factors for the energy density of fat and protein (9 and 4 kcal·g^−1^, respectively)^75^.

The chemical analysis consisted in determination of the mineral content in mineralized plant samples (n = 3) of each combination of vegetables. The contents of the elements were determined in 3-g vegetable samples after incineration in a muffle furnace at 480 °C. The resultant ash was solubilized on crucibles using 6 mol l^−1^ of spectrally pure hydrochloric acid (POCH, Poland). Na and K were analyzed using flame atomic emission spectroscopy (FAES) with a flame photometer (Pye Unicam SP 2900, Cambridge, UK). Ca, Mg, Zn, Cu, and Fe were determined using flame atomic absorption spectroscopy (FAAS) with a SOLAAR 939/959 spectrophotometer (Unicam, Cambridge, UK) according to the Polish Norm PN-EN ISO 6869:2002^76^. The phosphorus content was determined with the spectrometric method at 400 nm using a Helios Alpha UV–VIS apparatus (Spectronic Unicam, Leeds, UK), according to AOAC^74^. The detailed parameters of the chemical determinations were described in an earlier publication^77^. The content of Cd and Pb was determined using ICP (inductively coupled plasma mass spectrometry) in a Varian 820 MS spectrometer (Varian, Melbourne, Australia). Cd and Pb standards characterized by 99.999% purity and certified reference material (CRM): INCT-TL-1 Tea leaves (containing 0.030 mg of Cd and 1.78 mg of Pb per 1 kg) were used.

### Determination of vitamin C and A, carotene, and total phenols

The content of vitamin C in the analyzed vegetables was determined with the HPLC (Agilent Technologies, Model 1100, Waldbronn, Germany) technique and the method described by Valente et al.^78^ with minor modifications. The samples were placed in a ReproSilPur C18 column (250 × 4.6 mm, particle size: 5 µm; Dr. Masich, GmbH). The mobile phase contained 20 mM ammonium dihydrogen phosphate, pH 3.5, and 0.015% (w/v) metaphosphoric acid at a flow rate of 0.6 mL/min. Pure L-ascorbic acid was used as a standard.

Vitamin A was analyzed with the use of an Agilent 1100 series HPLC system (Wald-bronn, Germany) equipped with an Inertsil ODS-SP column (4.6 × 150 mm, 5 µm, Shimadzu). The mobile phase was 90% v/v methanol in water, and the flow rate was 0.8 mL/min. The absorption wavelength for detection was 325 nm, and the sample volume was 10 µl. The separation was carried out at the temperature of 30 °C.

For determination of carotene, the samples were eluted isocratically in the HPLC mobile phase at a flow rate of 1.2 mL/min (Waldbronn, Germany). A multiwave programmable detector (model MD 910, JASCO) and a Borwin PDA version 1.50 system controller (JASCO) were used. Carotenoids were identified and quantified at 450 nm against known standards. The details of the method are given in an earlier publication^5^.

The total phenolic content was determined using the spectrophotometric method^79^. A methanolic solution of the extract at a concentration of 1 mg/mL was used in the analysis. The reaction mixture was prepared by mixing 0.5 mL of the methanolic solution of the extract, 2.5 mL of the 10% Folin-Ciocalteu reagent dissolved in water, and 2.5 mL of 7.5% NaHCO_3_. The methodology of the total phenolic determination is described in previous publications^5,80^. The results were expressed as GAE (Gallic Acid Equivalents).

All analyses were performed in triplicate, and the means and standard deviations shown in the tables refer to the replication of fermentation within the vegetable species (n = 4) (each vegetable type was subjected to a separate fermentatiom process in 4 replications).

### Calculation of the dietary intake of nutrients in raw and fermented vegetables per serving for humans and daily demand coverage for animals

The daily human intake of proximal nutrients: protein, fat, energy, minerals: Ca, Mg, Zn, Cu, Fe, and vitamins C and A per one serving of the raw and fermented vegetables was calculated. One serving of the vegetables was assumed to weigh 100 g, based on the weight of vegetables recommended for consumption by the Department of Health and Human Services and the U.S. Department of Agriculture^17^. The determined values of nutrients in the vegetables were compared to the current dietary recommendations^17,18^.

Nutrients that are essential for the rearing cycle of monogastric animals (poultry and pigs) were analyzed: crude protein, ether extract, crude fiber, energy, and vitamin A as well as Na, Ca, Mg, P, and Zn minerals, for which the percentage coverage of the demand by a portion of 100 g dry matter of raw and fermented vegetables was calculated. Broiler ducks and fattener pigs in rearing period III (finisher), which can be fed with this type of fodder, were selected for the study as examples of animal species and technological groups. The calculations in these groups of animals were carried out in accordance with the nutritional recommendations developed by the Institute of Animal Physiology and Nutrition, Polish Academy of Sciences^19,20^.

### Statistical analysis

The analyses were performed in triplicate and all data were expressed as means. The normality of data and homogeneity of variances were tested using the Shapiro–Wilk and Brown-Forsythe tests, respectively. All data were subjected to multivariate analysis of variance (MANOVA) using general linear model (GLM) procedures with two fixed factors (raw and fermented vegetables). For MANOVA analysis, the Wilk test was used for evaluation of significant effects and one way analysis of variance (ANOVA) was used to determine significant differences between both groups and individual components at a confidence level of *P* < 0.05. All data were analyzed with the Statistica software version 13.3.

MANOVA was performed with the GLM procedure to test the main effects and their interaction. The following model was used:

$$ {\text{Y}}_{{{\text{ijk}}}}  ={\upmu } + {\text{A}}_{{\text{i}}}  + {\text{B}}_{{\text{j}}}  + {\text{AB}}_{{{\text{ij}}}}  + {\text{e}}_{{{\text{ijk}}}}  $$

where Y_ijk_—the measured variable, μ—the overall mean, A_i_—the effect of the ith species of the vegetables, B_j_—the effect of the jth fermentation process, AB_ij_—the interaction of A_i_ and B_j_, and e_ijk_—the random error.

ANOVA was used to determine significant differences between both groups and individual components. The following models were used:

$$ {\text{Y}}_{{{\text{ij}}}} \, = \, \mu \, + {\text{ a}}_{{\text{i}}} \, + {\text{ e}}_{{{\text{ij}}}} $$

where Y_ij_—the measured variable, μ—the overall mean; a_i_—the fermentation process effect, and e_ij_—the random error.

and

$$ {\text{Y}}_{{{\text{ij}}}} \, = \, \mu \, + {\text{ a}}_{{\text{i}}} \, + {\text{ e}}_{{{\text{ij}}}} $$

where Y_ij_—the measured variable, μ—the overall mean; a_i_—the vegetable species effect, and e_ij_—the random error.
